# The Simulation of Ester Lubricants and Their Application in Weak Gel Drilling Fluids

**DOI:** 10.3390/gels10030178

**Published:** 2024-03-03

**Authors:** Yao Dai, Fuwei Lu, Yuhua Tang, Yuanyuan Wang, Xinyi He, Tengfei Wang, Juan Wu

**Affiliations:** 1 College of Chemistry and Environmental Engineering, Yangtze University, Jingzhou 434023, China; 2021710203@yangtzeu.edu.cn (Y.D.); hxybjt999@126.com (X.H.); wangtengfeiforever@126.com (T.W.); wujuan9818@126.com (J.W.); 2 Hubei Engineering Research Centers for Clean Production and Pollution Control of Oil and Gas Fields, Yangtze University, Jingzhou 434023, China; 3 Petroleum Engineering Technology Research Institute of Sinopac Jiangsu Oilfield Branch Company, Yangzhou 225009, China; tangyuhua2000@126.com (Y.T.); wangyuanyuan0310@126.com (Y.W.)

**Keywords:** ester lubricants, methyl oleate, shear dynamics simulation, friction coefficient, weak gel drilling fluids

## Abstract

To enhance the performance and reduce the amount of ester-based lubricants used in weak gel drilling fluids, a shear dynamics simulation under extreme pressure conditions was employed to refine the formulation of the base oil and pressure additives. The simulation results were validated using fatty acid methyl, ethyl, and butyl esters. Fatty acid methyl ester demonstrated the lowest temperature increase and the highest load-bearing capacity post-shear. The four-ball friction test revealed that methyl oleate had a coefficient of friction of 0.0018, approximately a third of that for butyl oleate, confirming the simulation’s accuracy. By using methyl oleate as the base oil and oleamide as the pressure-resistant component, the optimal shear stress was achieved with a 10% addition of oleamide. A lubricant composed of 90% methyl oleate and 10% oleamide was tested and showed a coefficient of friction of 0.03 when 0.5% was added to bentonite slurry, indicating a strong lubricating film. Adding 1% of this lubricant to a low gel drilling fluid system did not affect its rheological properties, and the gel structure remained stable after seven days of aging. Field tests at the Fu86-3 well in the Jiangsu Oilfield of Sinopec confirmed that adding 1% of the ester-based lubricant to the drilling fluid significantly improved drilling efficiency, reduced drag by an average of 33%, and increased the drilling rate to 22.12 m/h. This innovation effectively prevents drilling complications and successfully achieves the objectives of enhancing efficiency.

## 1. Introduction

As global energy demands escalate, the exploration and extraction of oil and gas resources are shifting increasingly towards deeper and more intricate geological formations. Consequently, the necessity for drilling complex well structures, including high-displacement, extended-reach, and multilateral wells, is on the rise [1]. Oil-based drilling fluids are often used as the main wellbore working fluids from the open window sidetrack drilling to the completion of the horizontal section to ensure the downhole safety of complex structured wells [2]. Due to increasing environmental concerns and stricter regulations, there is a global shift towards replacing oil-based drilling fluids with water-based drilling fluids [3,4,5]. However, using water-based drilling fluids faces many realistic engineering complexities, such as the lubrication difficulties at the bottom of the well. This approach cannot easily meet the drilling requirements [6]. Researchers have developed various environmentally friendly and efficient water-based drilling fluid lubricants to improve the performance of water-based drilling fluids. Amongst them, modified vegetable oils and fats have been the focus of green lubricant development [7].

Although vegetable oils are environmentally friendly, they are less stable in alkaline environments than mineral oil lubricants. Moreover, vegetable oils are easily hydrolyzed and saponified at wellbore temperatures. It results in drilling fluid foaming, and the continuity of drilling fluid cannot be ensured [8]. The hydrolytically saponified vegetable oils also destroy the weak gel properties of drilling fluids [9]. Meanwhile, the cost of vegetable oil lubricants is much higher than that of mineral oil-based lubricants. In the field application process, the dosage of vegetable oil lubricants usually exceeds 3 *v*/*v*% of the drilling fluids, resulting in a high cost of use. If the cost-effectiveness ratio of vegetable oil lubricants is not solved, this type of treatment agent cannot be massively promoted in the field [10].

Researchers usually esterify vegetable oleic acid to balance the stability and lubrication performance of lubricants [11]. Haseeb et al. [12] investigated the effects of the length of carbon chains of esters on the lubrication and stabilization properties. The results showed that esters with different hydrophobic chains have good thermal stability. No thermal decomposition reaction occurred at 175–425 °C. The viscosity of all the ester lubricants does not decrease during the shear process. Li W. et al. [13] investigated the lubricants based on fatty acid methyl ester. A detailed comparison of the properties of fatty acid methyl esters and other vegetable oils showed that the reduction in extreme-pressure lubrication of the drilling fluids exceeds 70% in all cases when 2 wt.% fatty acid methyl esters are added to freshwater drilling fluids. The lubricants also have outstanding lubrication effects in seawater-based drilling fluids. Liu F. et al. [14] received several hydroxyl groups to ester lubricant. The extreme-pressure lubrication reduction rate and filter cake adhesion coefficient reduction rate can reach more than 80% after the hot rolling at 150 °C. Qian X. et al. [15] synthesized the environmentally friendly lubricant SMLUB-E by the esterification reaction. After being aged at 160 °C, SMLUB-E has a reduced molecular resistance coefficient from 0.47 to 0.05. SMLUB-E was applied in deep horizontal wells of the Taha Oilfield. No downhole failures, such as buttress, stuck drilling, and other downhole failures, were observed.

To improve the extreme-pressure lubrication performance, researchers focused on adding functional elements to the lubricants. The selected lubricant must have strong adsorption to the metal surface to survive in the harsh environment. The Sinopec Petroleum Engineering Research Institute used the S element cross-linked vegetable oil to form SMJH-1 [16]. The friction coefficient reduction rates before and after the hot rolling of SMJH-1 at a content of 1.0% in the base mud are 91.4% and 90.7%, respectively. The theoretical basis for this result is that the lubricating functional components containing sulfur, phosphorus, and other elements can form a protective film with a high strength on the surface of drilling tools, thereby improving the macroscopic lubrication effect. However, these elements also cause corrosion to the drilling tools. This phenomenon tends to cause downhole accidents. Furthermore, numerous solid nanomaterials have been used as extreme-pressure materials for lubricants. Liu Y. et al. [17] added expandable graphite to vegetable oil to form MVO-3. MVO-3 has excellent adsorption characteristics and lubrication properties. Modified expandable graphite has a composite lamellar structure. It can form elastic flake expansion in downhole conditions and generate dense expanded lubricant film by adsorption on a metal surface. Xiao J. et al. [18] added the hexagonal flake organic borate to the modified vegetable oil to form SDGL-2. SDGL-2 can withstand the temperature of up to 160 °C. When SDGL-2 is added at a dosage of 1.0%, the reductions in the lubricity coefficients of freshwater-based bentonite clay and saltwater-based bentonite clay are 85.33% and 77.51%, respectively.

In addition to the typical modified ester lubricants mentioned above, many commercial ester lubricant products can be found, all of which show good lubrication performance and compatibility with weak gel drilling fluids. However, the lubricant dosage is consistently higher than 2 *v*/*v*% in the field [19]. In some cases, the dosage of lubricants exceeds 5% [20]. Baker Hughes’ ROP enhancer is capable of reducing the friction coefficient of water-based drilling fluids to 0.17 at a field dosage of 2.7%, which is close to the level of 0.1 for oil-based drilling fluids [21]. In the context of unconventional oil drilling operations in the United States, a horizontal wellbore segment extending 7571 feet was successfully drilled utilizing a non-aqueous drilling fluid augmented with a 2.3% lubricant [22]. The introduction of this lubricant led to a marked reduction in frictional resistance, which decreased from levels exceeding 0.5 to approximately 0.35. Additionally, torsional friction experienced a reduction of 30–50%, while axial friction was diminished by 10–20%, demonstrating the efficacy of the lubricant in enhancing drilling performance. Reducing the cost of lubricant production and the amount of lubricant in the lubricant is still the main research direction of cost reduction and efficiency of environmentally friendly lubricants.

If ester lubricants can be made without the addition of expensive inorganic nanomaterials, the cost of lubricants is expected to be reduced. Thus, ester lubricants can be used on a large scale. Akaighe et al. [22] found that the horizontal portion of the complex wells is mainly controlled by the friction of boundaries and mixing layers. They also found that the lubricant has good stability, strong adsorption capacity on the metal surface, and high coverage density. A systematic study of the molecules defines the lubricant ‘head’ that adsorbs on the metal and the lubricant ‘tail’ that is densely arranged to provide a stable lubricant film, thereby providing insight into the optimal molecular structure. The contribution of each head group to the extreme-pressure lubrication performance is in the following order: ethyl ester < methyl ester < alcohol < amine. The carbon chain length of the tail group has little influence on the lubrication performance. Our group has also spent a long time simulating and applying vegetable oil-modified lubricants. Guo J. et al. [23] simulated the oil film thickness of vegetable oil lubricants on the surface of drilling tools by adsorption kinetics. Zhang S. et al. [24] simulated amide-modified vegetable oils and fats via restricted shear. They found that amide polar headgroups can be directionally adsorbed in the shear surface. This finding was confirmed by subsequent shear friction wear experiments. The understanding of tribology from other industrial applications can also be used in designing molecules to provide optimal lubrication in weak gel drilling fluids. Wang W. [25] modeled the effect of the poly-a-olefin molecular structure on lubrication performance using band pressure-confined shear and concluded that PAO molecules with long side-chains or with a specific form of side-chain distribution can be aligned in a well-ordered manner along the direction of shear to improve extreme-pressure lubrication performance under the ultimate pressure of 500 MPa. This model is close to the state of shear friction in horizontal wells and can be used to simulate the lubrication performance of ester lubricants under extreme pressure conditions. The molecular simulation is used to optimize the extreme-pressure lubrication performance of ester lubricants through the organic combination of the previous research foundation and the pressure-constrained shear model to obtain high-efficiency lubricants with a reasonable raw material price and low on-site dosage.

## 2. Results and Discussion

### 2.1. Shear Simulation and Model Validation of Ester Base Oil

Shear dynamics under extreme pressure were used to simulate the motion state of ester base oils between Fe shear surfaces. The effects of different molecular structures on these shear parameters were compared to optimize the base oil. The simulation accuracy was verified using four-ball friction and wear experiments with ester lubricants corresponding to the simulation.

**Figure 1:**
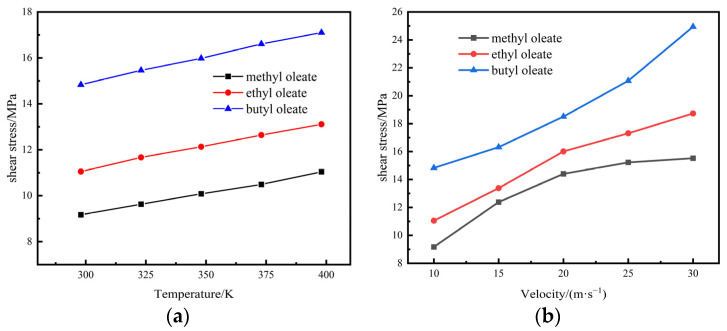
Average shear stress of base oils under different conditions: (**a**) different initial temperatures; (**b**) different shear speeds.

The magnitude of the friction generated between the lubricant and the shear surface can be reflected by the magnitude of the shear stress. The shear stress in the direction of the shear motion of the lubricant can be obtained by calculating the shear stress component $\tau_{xz}$ to which the lubricant molecules are subjected between the two layers of iron. During the shear simulation, the molecules were subjected to a combination of wall shear action and internal thermal motion of the fluid molecules. The shear stress of the lubricant fluctuates at the microscopic level. The average of the shear stresses was taken to represent the shear stress of the lubricant at the macroscopic level. The shear stresses of the three ester lubricant base oils at different initial temperatures and shear rates are shown in Figure 1.

Figure 1a shows that the shear stress gradually decreases as the number of carbon atoms on the head group in the ester lubricant decreases at the same initial temperature. Amongst the three ester base oils, the shear stress of methyl oleate is the lowest, approximately one-half of that of butyl oleate. This finding indicates that methyl oleate possesses low shear stress. Under different shear temperature conditions, the shear stress of methyl oleate was significantly lower than that of the two other base oils. The results indicate that methyl oleate has good lubricity at different temperatures. The average shear stresses of the three ester lubricants at different shear speeds are shown in Figure 1b. Figure 1b shows that under the same shear rate condition. The shear stress gradually decreases as the number of carbon atoms on the head group in the molecule decreases. With the increasing shear rate, the shear stresses of methyl oleate become significantly lower than those of the other oleates. The results indicate that methyl oleate has good lubricity under different shear rate conditions. The lubricating properties of the base oils become increasingly prominent as the number of carbon atoms in the head group decreases. Moreover, the lubricating properties of methyl oleate are notable.

**Figure 2:**
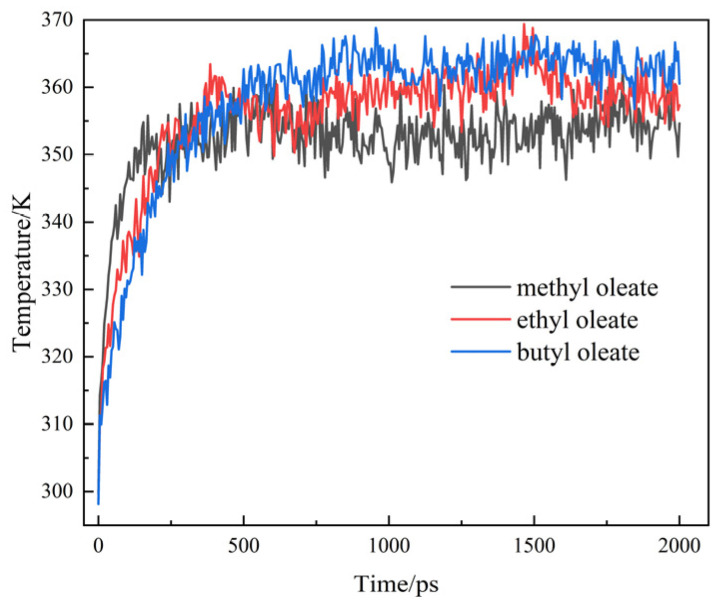
Variation of mean temperature of ester base oils with shear time.

The temperature change of the fluid during shear is directly associated with the lubricating performance of the fluid. In the case of prolonged friction, the frictional heat leads to an increase in the fluid temperature. This thermal alteration can significantly compromise the material’s lubricating performance. Figure 2 shows the variation of the average temperature of the ester base oil with shear time.

Figure 2 illustrates the temperature dynamics of the three systems during the shear process. Initially, all systems experience rapid heat generation, leading to a significant increase in temperature. Subsequently, the systems gradually approach thermal equilibrium. The equilibrium time is approximately 500 ps. Compared with the steady state temperature of the two other base oils, the equilibrium temperature of methyl oleate is approximately 350 K, indicating a low equilibrium temperature. Under the same shear rate and initial temperature conditions, the difference in shear temperature after equilibrium is caused by the lubricant film strength [26]. The difference in lubricant film strength of different lubricants can be reflected by the oil film density at the shear surface.

**Figure 3:**
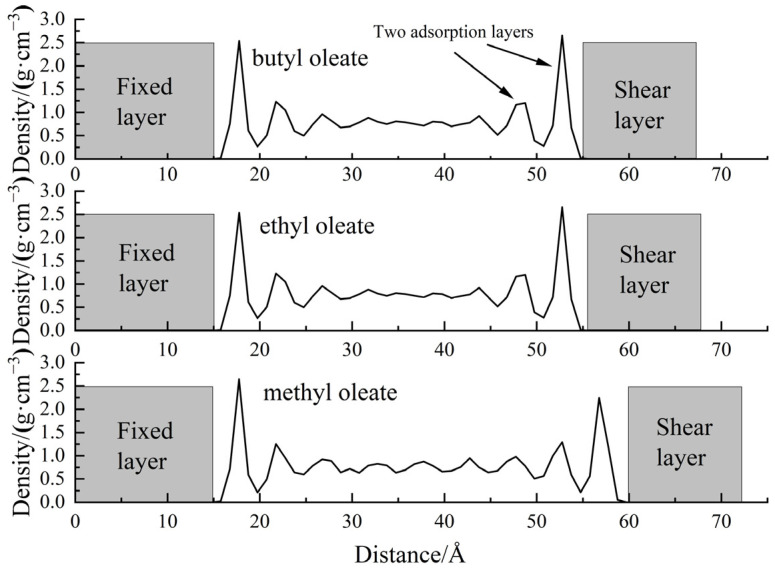
Density distribution profile of ester lubricants after shearing.

The density distribution of the base oil at different positions between the shear surfaces and the difference in shear stress were analyzed. The post-shear density distribution profiles of the three ester base oils are shown in Figure 3.

Figure 3 shows that the three ester lubricants have the highest spikes near the two iron surfaces, and the peaks are approximately 5 Å wide. Two spikes are significantly higher than the density in the middle on both iron surfaces. This finding indicates that the molecules of the ester lubricants are adsorbed on the iron surfaces, and the adsorbed layers of the three lubricants are all composed of two molecular layers. Under a pressure of 500 MPa, the density of methyl oleate without contact with the shear surface was 0.81 g·cm^−3^, remaining consistent with the density at ambient pressure. High pressure did not affect the bulk density of methyl oleate. The density of the oil film in contact with a solid surface was 2.59 g·cm^−3^, which is three times the bulk density. The compactness of the oil film at the shear surface is significantly higher, being a primary reason for the formation of a stable oil film by ester lubricants on friction surfaces. Among the three types of ester-based base oils, methyl ester possesses the highest oil film density, while ethyl ester has a density of 2.48 g·cm^−3^, and butyl ester has a density of only 2.3 g·cm^−3^. The oil film density of methyl ester is significantly greater than the other two, indicating that it has superior film strength. This may be attributed to the fact that among the three ester-based lubricants, methyl ester has the smallest carbon number in its head group and is more polar. This results in methyl ester having a superior adsorption capability on the shear plane compared to the other two esters.

**Figure 4:**
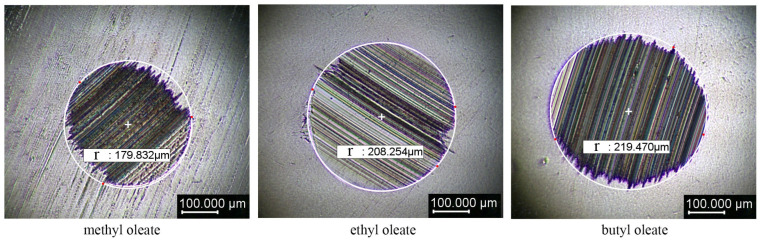
Friction test wear spot size of ester lubricants.

The antiwear properties of the three ester lubricants corresponding to the simulation were tested by the four-ball friction test. The test procedure was performed according to the SH/T 0189-92 method. Methyl oleate, ethyl oleate, and butyl acetate were tested. The friction spot radius is shown in Figure 4.

**Figure 5:**
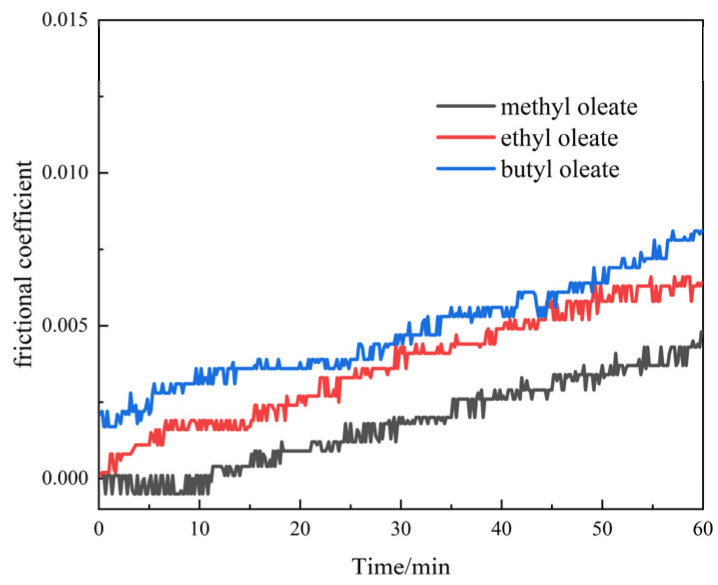
Coefficient of friction of ester lubricants over time.

Figure 4 shows that the friction spot radius increases sequentially with the increase in the length of the carbon chain of the head group of the oleic acid ester materials. The mottle radius of methyl oleate is 179.83 μm, whereas that of butyl oleate reaches 219.47 μm. The results show that the lubricant’s protective effect on the friction area of the metal weakens with the increase in the length of the head-group covetousness of oleate esters. The friction coefficients of the three oleate esters during the test time of 60 min are shown in Figure 5.

Figure 5 shows that the friction coefficients of methyl oleate, ethyl oleate, and butyl oleate decreased sequentially at the same time point. The change rules of the friction coefficient are the same in 60 min test time. The results show that methyl oleate is more capable of reducing the friction coefficient of the shearing process and has better anti-friction performance than the two other oleic acid esters. The pattern of simulation results is consistent with the experimental results. This finding is the main reason why most of the ester lubricants in the market choose methyl oleate as a lubricant.

### 2.2. Selection of Extreme-Pressure Components and Simulation of Efficient Lubricant

Methyl oleate is a commonly used drilling fluid lubricant in the market, and the dosage in the drilling fluid usually exceeds 3%. An extreme-pressure functional component with a low dosage in the lubricant was selected and mixed with methyl oleate to realize the reduction and increase the efficiency of methyl oleate in drilling fluids by improving the lubrication effect and reducing the dosage of the lubricant. This approach is an economic choice.

**Figure 6:**
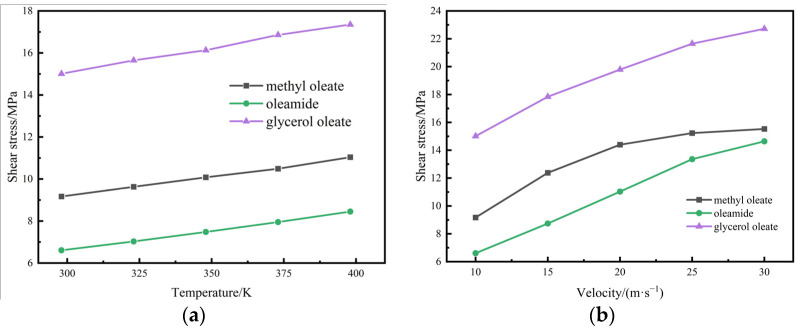
Mean shear stresses of extreme-pressure functional components: (**a**) different initial temperatures; (**b**) different shear speeds.

Choosing expensive nano solid extreme-pressure materials to improve lubrication performance is slightly economical because oleate derivatives can form high-density oil films on shear surfaces. Oleic acid-modified extreme-pressure lubricants are good choices in terms of performance and economy. Glycerol oleate, containing multiple hydroxyl groups, and oleic acid amide, containing amine groups, were selected for shear dynamics simulation to investigate the extreme-pressure lubrication performance of oleic acid-modified materials with different polar head groups. Figure 6 shows the average shear stresses of the extreme-pressure functional components and methyl oleate at different initial temperatures and initial shear speeds.

Figure 6 shows that the shear stress of oleate is higher than that of methyl oleate under the same shear conditions. Moreover, oleamide has a significantly lower average shear stress than methyl oleate. This finding suggests that glyceryl oleate may not be suitable as an extreme-pressure lubrication component. Oleamide is more suitable than glyceryl oleate to be added to methyl oleate.

**Figure 7:**
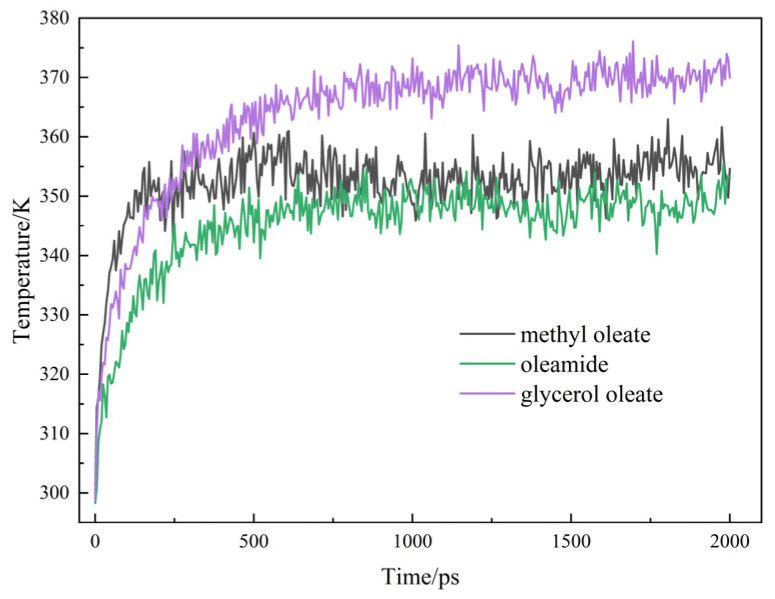
Average shear temperature of extreme-pressure lubrication component fluids.

Figure 7 shows the average shear temperatures of the two extreme-pressure functional components as a function of shear time.

Figure 7 shows that the kinetic equilibrium time between the two functional components at the shear surface is the same as that of the ester base oil, which is approximately 500 ps. The fluid shear temperature reaches equilibrium after 500 ps. The post-equilibrium shear temperature of glycerol oleate is 370 K. Oleamide has a lower equilibrium temperature of approximately 345 K. Oleamide is suitable for use as an extreme-pressure lubricant additive.

**Figure 8:**
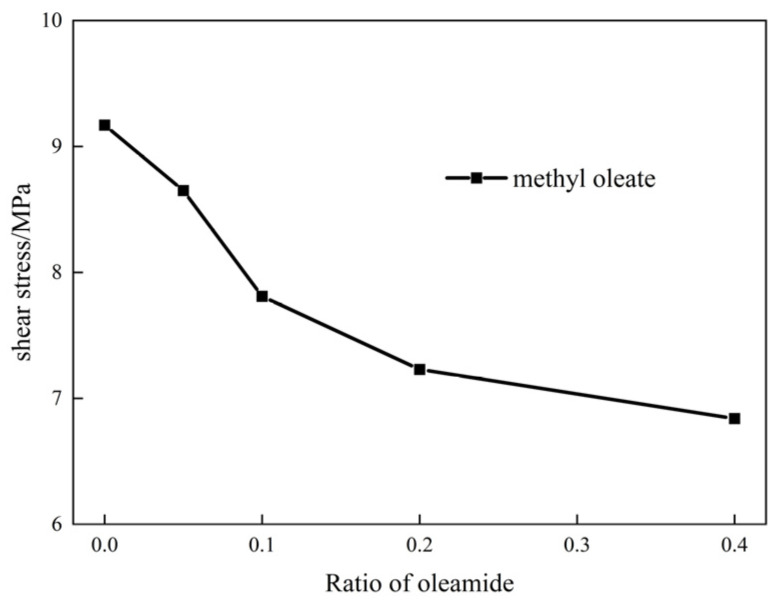
Average shear stress of two-component mixtures of methyl oleate and oleamide.

Methyl oleate was used as the base oil, whereas oleamide was used as the extreme-pressure lubrication additive. The two components were mixed to form a lubricant. The effects of different oleamide additions on the lubricant performance were investigated via shear dynamics simulation. Under the same shear conditions, the average shear stress of the two-component mixtures in the simulation varies with the oleamide content, as shown in Figure 8.

Figure 8 shows that the shear stress of the mixtures decreases significantly with the addition of oleic acid amide to methyl oleate. This finding indicates that the adsorption capacity of the amide group on the iron surface is stronger than that of the oleate base oil. The maximum decrease in shear stress of the mixtures was observed when the amount of oleamide was added up to approximately 10%. The shear stress drop of the mixture tends to be moderated when the content of oleamide exceeds 10%. Given the lubrication performance and economy of the mixtures, the mixture with 10% oleamide content was selected for performance evaluation.

The above simulations of ester-based lubricant structures and compositions are based on the same hydrophobic tail group, varying the polar head group for optimization simulations. The method has been able to screen out bio-lubricants with outstanding performance. The guiding role of computational simulation in the macroscopic field will be more prominent if it can be coupled with the test conditions of the drilling fluid, and the relationship between the structure of the lubricant and the test conditions can be optimized comprehensively by the response surface method [27]. The related research will be strengthened in our subsequent studies.

### 2.3. Performance Testing of Ester Lubricants

**Figure 9:**
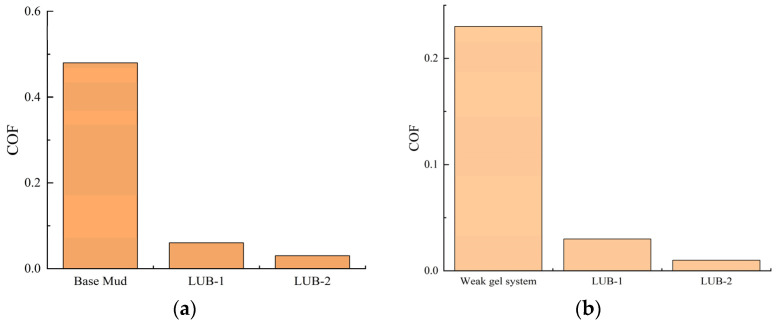
Lubricity factor of the test system with 0.5% lubricant: (**a**) base mud; (**b**) weak gel drilling fluid.

Methyl oleate was named LUB-1. A mixture of methyl oleate with 10% oleamide was named LUB-2. The lubrication performances of LUB-1 and LUB-2 were tested for their lubricating properties in base stock and weak gel drilling fluids, respectively. The results of the lubrication performance are shown in Figure 9.

Figure 9 shows that the lubricity coefficient of the base mud is 0.47 after hot rolling at 120 °C for 16 h. After adding 0.5% lubricant LUB-1, the lubricity coefficient is reduced to 0.06, and the reduction rate of the lubricity coefficient is 87.5%. Under the same conditions, the lubrication coefficient of LUB-2 is reduced by 93.75%. In the weak gel drilling fluid, the lubricity coefficient of the drilling fluid without lubricant is 0.23. After the addition of 0.5% lubricant LUB-1, the lubricity coefficient is 87.0%, and the lubricity coefficient reduction rate with LUB-2 is 95.65%. The results show that the lubrication performance of methyl oleate with the addition of oleamide is significantly better than that of methyl oleate in the base mud and drilling fluid.

The effect of lubricant on the foaming and viscosity of the base mud are shown in Table 1.

Table 1 shows that the viscosity of the base mud increases significantly after the addition of LUB-1, and the density decreases. However, the effect of LUB-2 on the rheological properties of the base mud is minimal, and the viscosity increase is smaller. The reason is that after the addition of LUB-1 to the base mud, the base mud is mixed with a large number of air bubbles under stirring conditions, resulting in a decrease in fluid density and an increase in viscosity. The blistering of the base mud after hot rolling has a prominent disadvantage. When LUB-2 is added to the matrix, the density and viscosity of the matrix do not change significantly before and after hot rolling. The results show that mixing 10% oleamide in methyl oleate can effectively inhibit the foaming property of methyl oleate on the base stock and avoid the construction difficulties caused by the foaming of the drilling fluid. The effect of 0.5% lubricant on the performance of weak gel drilling fluid before and after hot rolling at 120 °C was tested. The results are shown in Table 2.

Table 2 shows that after hot rolling at 120 °C, the apparent viscosity and plastic viscosity of the weak gel drilling fluid system with the addition of LUB-1 increase significantly by approximately 2 mPa·s. This increase is caused by the foaming of fatty acid methyl ester in the drilling fluid. Before and after the addition of LUB-2, the viscosity of the drilling fluid decreases by more than 1 mPa·s, and the filtration loss decreases. This result indicates that LUB-2 does not affect the rheology of the drilling fluid and assists in decreasing the filtration loss of the drilling fluid. The experimental results show that LUB-2 is compatible with weak gel drilling fluid.

Another disadvantage of ester lubricants is that they are easily hydrolyzed under alkaline conditions. The formation of oleic soaps as by-products causes foaming of the drilling fluid and a reduction in lubricating properties. The pH of the weak gel drilling fluid system was set at 8.0, consistent with the pH of the drilling fluid in the field. After the addition of 1% LUB-2 to the drilling fluid, the drilling fluid was aged at 120 °C for 7 days to investigate the long-lasting performance of LUB-2. The results are shown in Table 3.

Table 3 shows that when 1% of weak gel drilling fluid is added for 1 day of hot rolling, the apparent viscosity, plastic viscosity, and shear force are reduced, and the density is reduced by 0.01 g·cm^−3^ because of the self-adjustment of the gel structure before and after hot rolling. The change rule of viscosity is consistent with that in Table 2. In the later stage of the hot rolling process lasting 7 days, the viscosity of the drilling fluid system changes by less than 1 mPa·s. Moreover, the gel structure of the drilling fluid has not changed fundamentally. The density of the drilling fluid does not change during the continuous hot rolling process, indicating that LUB-2 is not hydrolyzed during the hot rolling process and can resist the hydrolytic erosion effect at pH 8. This experiment provides support for the future field application of LUB-2.

### 2.4. Field Application of LUB-2

LUB-2 was applied on-site in the Hua3-01 well. The well was completed at a depth of 3160 m, with a slope of 42 degrees and a horizontal displacement of 1194 m. Then, 1% LUB-2 was added during open-window sidetrack drilling. The performance of the drilling fluids at different well depths is shown in Table 4.

Table 4 shows that the viscosity of the field drilling fluid is greater than that of the laboratory-formulated drilling fluid in Table 3 at the same density because of the intrusion of formation clay into the drilling fluid. At 2337 m well depth, the drilling resistance is 15 tonnes before the addition of 1% LUB-2. After the addition of LUB-2, the drilling resistance decreases to 10 tonnes at 2548 m well depth, with a 33% reduction in frictional resistance. The resistance remains unchanged at 10 tonnes during subsequent drilling until completion. After the addition of the lubricant, the apparent viscosity and plastic viscosity of the drilling fluid are reduced by 2.5–3.0 mPa·s, which attenuates the adverse effect on drilling due to the increase in clay viscosity. The results show that methyl oleate mixed with amide can effectively reduce the friction resistance of drilling wells and ensure smooth drilling with less than 1% dosage. LUB-2 achieved the same effect as a 3% dosage of traditional ester-based lubricants when used at a 1% dosage. Thus, the economy of ester lubricants can be improved, and the application prospect of ester lubricants can be expanded.

## 3. Conclusions

The conclusions of this work are as follows.

(1) Methyl oleate and oleamide are selected as the base oil of the ester lubricant and the extreme-pressure lubricant additive through molecular dynamics shear simulation under extreme pressure. The ratio of oleamide and methyl oleate is optimized, and the ester lubricant LUB-2 is formed when 10% oleamide is added to methyl oleate. LUB-2 can effectively balance the lubricating performance and cost of the lubricant.
(2) The results of the evaluation of the lubricant show that the lubricity coefficient of the drilling fluid is reduced by 95.65% when 0.5% LUB-2 is added to the weak gel drilling fluid. This reduction is more than the reduction in most of the ester lubricants in the market. LUB-2 is not hydrolyzed in the weak gel at pH 8.0 and is compatible with the drilling fluid. This feature does not affect the gel performance of the drilling fluid.
(3) The results of the field application show that LUB-2 can reduce the friction resistance by 33% when 1% of LUB-2 is added to the drilling fluid in the field, and the lubrication performance remains unchanged after a total horizontal displacement of 1194 m from the opening of the window side drilling to the completion of the well. The addition of 10% oleic acid amide to methyl ester can greatly improve the economy of ester lubricants and increase the application prospect of ester lubricants.

## 4. Materials and Methods

### 4.1. Shear Simulation of Ester Lubricants

#### 4.1.1. Structure Models

**Figure 10:**
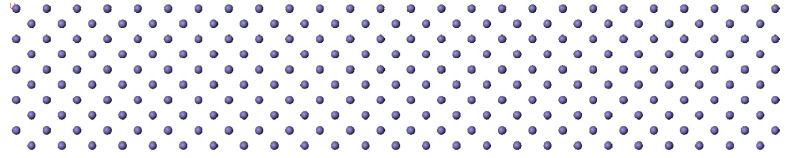
The model of Fe(0 0 1).

**Figure 11:**
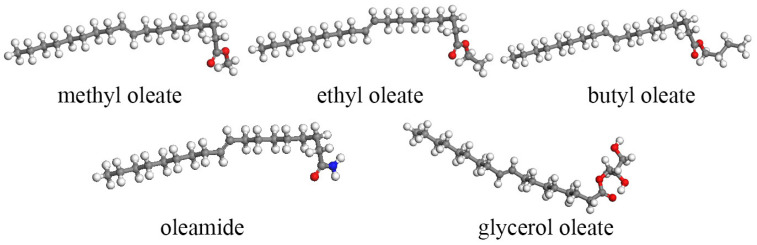
The model of component structure models of lubricants. (Red represents O; Blue represents N).

The establishment of a suitable lubricant base oil model has a convincing influence on the accuracy of the molecular dynamics simulation results. The surface of the drilling tools in the drilling process is dominated by iron. The Fe(0 0 1) surface was used as the surface model of the drilling tools to maintain continuity with the previous study [23,24]. As shown in Figure 10, the ‘sandwich model’ of Fe-lubricants-Fe was constructed, with the size of the upper and lower layers of Fe being 80.26 Å × 40.13 Å × 14.4 Å. The single-component structure models of the five lubricants are shown in Figure 11. They are the same as those of the lubricant base oils.

**Figure 12:**
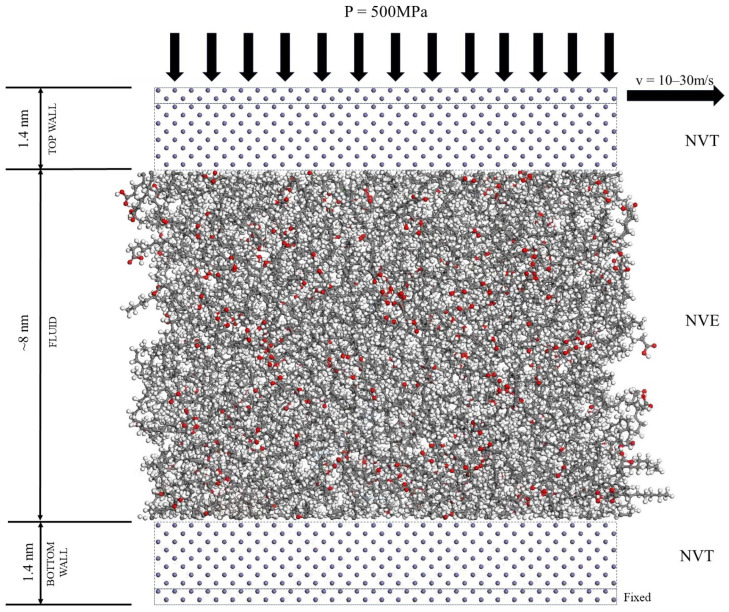
Shear simulation of the “sandwich model”. (Red represents O).

After the lubricant molecules and Fe(0 0 1) surfaces were constructed, the model was optimized in terms of structure and energy with a pcff force field. Fine accuracy, an NVT system, a temperature of 298 K, a step size of 1.0 fs, and a simulation time of 50 ps are input into the structural optimization simulation. The lubricant system was constructed to contain 200 lubricant molecules. A ‘sandwich model’ was constructed using two layers of Fe(0 0 1) surfaces with the lubricant system. Van der Waals and Coulomb force interactions were calculated using the atom-based and Ewald methods with a truncation radius of 1.55 nm. The initial structure of the ‘sandwich model’ is shown in Figure 12.

#### 4.1.2. Shear Simulation of Single Lubricant Structure

**Figure 13:**
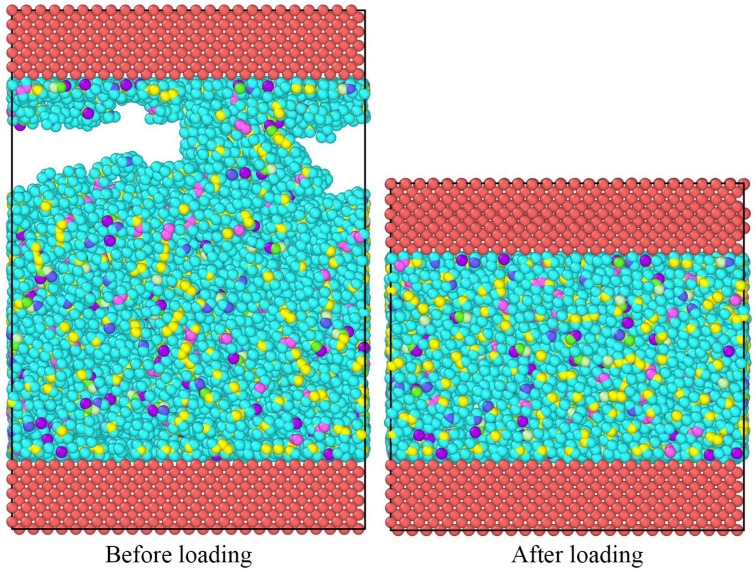
Structural changes of the “sandwich model” before and after loading.(Red represents Fe; Blue, Purple and Pink represents different C; Cyan represents H; Green represents O;Yellow represents N).

After the model was built, the shear simulation was performed according to the following steps:

(1) Structural optimization: After the establishment of the ‘sandwich model’, the positions of the upper and lower iron layers were fixed, and molecular dynamics simulations were used to optimize the overall structure of the lubricant system in the middle layer.
(2) Load process: The simulation system adopted the NVT system, fixed the lower iron wall, and applied a pressure of 500 MPa to the upper iron wall. The system’s temperature was set to 298 K, the step size was 1.0 fs, and the simulation time was 50 ps, thereby allowing the system to reach a stable state [25]. The structural changes in the ‘sandwich model’ before and after loading are shown in Figure 13.
(3) Shear process: After the completion of the load iron wall surface, the system continued using the NVT system, namely, the lubricant system using the NVE system, to simulate the lubricant in the shear process and produce the shear heat phenomenon [28,29]. The NVT ensemble is a canonical ensemble, which is a collection of systems with the same number of molecules N, the same volume V, and the same temperature T, represented by the symbol (N, V, T). The NVE ensemble is a microcanonical ensemble, which is a collection of systems with the same number of molecules N, the same volume V, and the same energy E. The lower iron wall surface was fixed, and the upper iron wall surface of the upper-most layer of iron atoms was set at a speed of 10–30 m/s along the x-axis direction of the shear. The initial temperature of the system was set to 298–398 K. The step size was 1.0 fs, and the simulation time was 2000 ps.

#### 4.1.3. Shear Simulation for Lubricant Structure Optimization

The optimized oleic acid esters were used as base oils. The oleic acid amides containing amine groups and oleic acid glycerides containing hydroxyl groups were used as the extreme-pressure functional components. The base oils were compounded with the extreme-pressure agents to form new lubricants. The shear simulations are conducted according to the above method. The effects of different dosages on the performance of the systems were also compared. The extreme-pressure lubricant component was preferred, and the extreme-pressure lubricant component and base oil were compounded to optimize the structure of the hybrid lubricant through shear simulation under extreme pressure.

### 4.2. Experimental Materials and Apparatus

The materials used in this study include ethyl oleate, ethyl oleate, butyl oleate, oleic acid amide, monoglyceride oleate, and anhydrous ethanol. The apparatuses used include a four-ball friction tester with steel balls and the orthogonal metallurgical microscope PH-M3230.

### 4.3. Friction and Wear Experiment

The antiwear performance of the lubricant was tested using the SGW-10G, a four-ball friction tester with the lever. The four-ball friction test with lever refers to the industry standard [30]. Before the experiment, anhydrous ethanol was used to clean the instrument parts to ensure that the lubricant was not contaminated by impurities on the instrument’s surface. A load of 147 N was applied to the steel balls at room temperature. The temperature of the test chamber was increased to 75 °C, and the top ball was rotated at 1200 r/min for 60 min. The coefficient of friction was measured during the test. At the end of the test, the size of the wear spots on the ball was observed using an orthogonal metallurgical microscope.

### 4.4. Evaluation of Lubrication Property in Weak Gel Drilling Fluids

#### 4.4.1. Preparation of Drilling Fluids

(1) Base mud

Water, bentonite, and sodium carbonate were mixed according to the mass ratio 400:20:1. The parameters are consistent with the SINOPEC standard [31]. They were stirred at 6000 r/min for 30 min, sealed, and placed at room temperature for 24 h.

(2) Weak gel drilling fluid

The composition of the weak gel drilling fluid formulation was 0.3% polymer FA367, 0.8% polyacrylonitrile–polyacrylamide ammonium composite salt, 1% potassium polyacrylate KPA, 3% of the reduction of filtration loss agent QS-4, 1.5% amphiphilic pressure-bearing plugging agent, 1.5% environmental protection nano blocking agent YRD-BA01, and 0.2% caustic soda. The aggravating agent barite was mixed in the base mud and aggravated to 1.2 g/m^3^. The weak gel drilling fluid was mixed at 6000 r/min for 30 min, sealed, and placed at room temperature for 24 h.

#### 4.4.2. Evaluation of Lubrication Performance

(1) Evaluation of extreme-pressure lubrication performance

According to the technical indexes and test methods of the Sinopec enterprise standard ‘Technical Requirements of Liquid Lubricant for Water-based Drilling Fluid’ (Q/SH CG0004-2023), FANN 21200 extreme-pressure lubrication instrument was used to test the lubrication coefficient of the base mud, with 0.5% lubricant being added to the slurry (the test condition was hot rolling for 16 h at 120 °C).

(2) Determination of drilling fluid compatibility

The lubricant was added directly into the prepared base mud or polymer drilling fluid and mixed at high speed for 20 min to make the lubricant evenly dispersed. According to the Chinese national standard GB/T 16783-1997 ‘Field Test Procedures for Water-based Drilling Fluids’, the viscosity, filtration loss, and rheological parameters of the base mud or polymer drilling fluid were measured to investigate the compatibility of the lubricant with the polymer drilling fluid.

(3) Performance test after a long time of aging

The lubricant was added into the polymer drilling fluid, stirred well at high speed for 20 min, and aged continuously at 120 °C for 7 days. The data of the drilling fluid were tested every day to investigate the effect of the lubricant on the long-time gel performance of the drilling fluid.

## Figures and Tables

**Table 1:** The impact of lubricants on the performance of base mud.

	Test Conditions	AV/ mPa·s	PV/ mPa·s	YP/ Pa	FL_API_/mL	ρ/ (g·cm^−3^)
Base mud	before hot rolling	7.0	6.0	1.0	23.2	1.03
	120 °C 16 h	7.5	5.5	2.0	28.4	1.03
+0.5% LUB-1	before hot rolling	10.0	7.0	3.0	18.9	0.98
	120 °C 16 h	9.0	6.5	2.5	24.5	0.95
+0.5% LUB-2	before hot rolling	7.5	6.0	1.5	18.5	1.025
	120 °C 16 h	8.5	6.5	2.0	23.9	1.02

**Table 2:** The impact of lubricants on the performance of weak gel drilling fluids.

	Test Conditions	AV/ mPa·s	PV/ mPa·s	YP/ Pa	FL_API_/ mL
Weak gel drilling fluid	before hot rolling	43.0	35.0	8.0	2.8
	120 °C 16 h	36.5	32.0	4.5	3.4
+0.5% LUB-1	before hot rolling	46.0	38.5	7.5	2.6
	120 °C 16 h	38.0	34.0	4.0	3.0
+0.5% LUB-2	before hot rolling	42.0	35.0	7.0	2.4
	120 °C 16 h	34.5	31.0	3.5	3.1

**Table 3:** The influence of lubricants on the stability of weak gel drilling fluids.

Test Conditions	Gel/Pa		AV/mPa·s	PV/mPa·s	YP/Pa	ρ/(g·cm^−3^)
	10 s	10 min				
before hot rolling	3	6	42.0	35.0	7.0	1.20
Day 1	1.5	4.5	34.5	31.0	3.5	1.19
Day 2	1.5	4	34.0	31.0	3.0	1.19
Day 3	1.5	4.5	34.0	30.5	3.5	1.19
Day 4	1.5	4.5	33.0	30.5	2.5	1.19
Day 5	2	4	33.5	30.5	3.0	1.19
Day 6	1.5	4	33.0	30.5	2.5	1.19
Day 7	1.5	4	33.0	30.0	3.0	1.19

**Table 4:** Drilling fluid performance in Hua3-01 well.

Well Depth/ m	AV/ mPa·s	PV/ mPa·s	YP/ Pa	FL_HTHP/120 °C_	Drilling Resistances/T	Note
2143	47	33	14	14.8	14	Without Lubricants
2337	50	35	15	14.0	15	Adding 1% LUB-2
2428	47.5	33	14.5	12.8	10	
2713	48.5	33	14.5	14.2	10	
3160	48.5	33	14.5	14.0	11	

## Data Availability

The original contributions presented in the study are included in the article. Further inquiries can be directed to the corresponding author/s.

## References

[1] Sonmez A., Kok M.V., Bal B., Bagatir G., Gucuyener I.H. (2021). Comprehensive Approach to Torque and Lost Circulation Problems in Geothermal Wells in Terms of Drilling Fluid. Geothermics.

[2] Zhang Y., Mian C., Jin Y., Lu Y., Liang C., Wang D., Du X., Li W. The Influence of Oil-Based Drilling Fluid on the Wellbore Instability and Fracturing in Complex Shale Formation. Proceedings of the 50th U.S. Rock Mechanics/Geomechanics Symposium.

[3] Rojas D., Soruco A., Jimenez C., Soriano V., Ahmed R. High Performance Water-Based Fluid Replaces Oil-Based Mud on the Execution of a Complex 3D Well Trajectory. Proceedings of the SPE Latin American and Caribbean Petroleum Engineering Conference.

[4] Heikal A., Banna M.E., Manescu G., Mulaifi M.A., Mohammed I. High-Performance Water-Base Fluid Performs as an Environmentally Friendly Alternative to Oil-Base for Drilling Challenging. Proceedings of the SPE Asia Pacific Oil and Gas Conference and Exhibition.

[5] Dye W., Daugereau K., Hansen N.A., Otto M.J., Shoults L., Leaper R., Clapper D.K., Xiang T. (2006). New Water-Based Mud Balances High-Performance Drilling and Environmental Compliance. SPE Drill. Complet..

[6] Liu J., Li G., Xia Y. (2020). Technical Progress on Environmental-Friendly, High-Performance Water-Based Drilling Fluids. Environ. Earth Sci. Res. J..

[7] Zhang L., Hou S., Wu Y., You F., Zhang J. (2022). Research Progress and Development Trend of Environmentally Friendly Lubricants for Drilling Fluids. Oilfield Chem..

[8] Kania D., Yunus R., Omar R., Abdul Rashid S., Mohamad Jan B. (2015). A Review of Biolubricants in Drilling Fluids: Recent Research, Performance, and Applications. J. Petrol. Sci. Eng..

[9] Amanullah M., Ramasamy J., Alouhali R. HSE-Friendly Lubricants to Safeguard Environment and Enhance Operational Excellence. Proceedings of the International Petroleum Technology Conference.

[10] Zhao X., Li D., Zhu H., Ma J., An Y. (2022). Advanced Developments in Environmentally Friendly Lubricants for Water-Based Drilling Fluid: A Review. RSC Adv..

[11] Raof N.A., Hamid H.A., Aziz N., Yunus R. (2022). Prospects of Plant-Based Trimethylolpropane Esters in the Biolubricant Formulation for Various Applications: A Review. Front. Mech. Eng..

[12] Nehal S. (2023). Ahmed Citric Acid-Based Esters as Potential Synthetic Lubricants: A Study of Their Synthesis, Rheological Properties and Thermal Stability. Tribol. Trans..

[13] Li W., Zhao X., Peng H., Guo J., Ji T., Chen B., You Z., Liu L. A Novel Environmentally Friendly Lubricant for Water-Based Drilling Fluids as a New Application of Biodiesel. Proceedings of the IADC/SPE Asia Pacific Drilling Technology Conference.

[14] Liu F. (2019). Study on Mechanism of Shear Response Gel and Strong Adsorption Water-Based Lubricant. Ph.D. Thesis.

[15] Qian x., Xuan Y., Lin Y., Yang X. (2020). Development and Application of an Environmental-Friendly Drilling Fluid Lubricant SMLUB-E. Pet. Drill. Tech..

[16] Dong X., Wang L., Yang X., Lin Y., Xue Y. (2015). Effect of Ester Based Lubricant SMJH-1 on the Lubricity Properties of Water Based Drilling Fluid. J. Petrol. Sci. Eng..

[17] Liu Y., Qiu Z., Zhong H., Meng M., Zhao X., Nie Z., Huang W. Development of a Novel Anti-Temperature, Anti-Wear and Ecofriendly Lubricant SDL-1 for Water-Based Drilling Fluid. Proceedings of the International Petroleum Technology Conference.

[18] Shan K., Qiu Z., Zhang W., Zhong H., Zhao X., Mo J. (2021). Preparation and Performance Evaluation of an Environmentally Friendly Lubricant for Water-Based Drilling Fluid. IOP Conf. Ser. Earth Environ..

[19] Amanullah M. Coefficient of Friction Reducing Efficiency of ARC Eco-Lube. Proceedings of the IADC/SPE Asia Pacific Drilling Technology Conference.

[20] Fereidounpour A., Mohammad H., Hosseini M. (2020). A Field Study, Laboratory Test and Cost Estimation of Solid and Liquid Lubricants in Directional Wells to Reduce Friction Coefficient and Improve ROP. SN Appl. Sci..

[21] Everhard I., Willis S., Villalobos M., Clapper D., Salem H.A., Hughes B. Reduced Drilling Days and Low Friction Factors Hallmark Eagle Ford Water-Based Fluid Performance. Proceedings of the 2014 AADE Fluids Technical Conference and Exhibition.

[22] Akaighe N., Zeilinger S., Cutler J., Bhandari D., Bunquin J., Bharadwaj N. Low Friction Drilling Fluid Additive Technology. Proceedings of the SPE/IADC International Drilling Conference and Exhibition.

[23] Guo J., Zhou J., Lu F., Li Y., Wang Z., Huang C., Tian Z., Kong Y. (2018). Simulation of Adsorption of Lubricant Base Oil on the Surface of Drilling Tools. Drill. Fluid Complet. Fluid.

[24] Zhang S., Dai Y., Xu H., Wang J., Lu F., Liu G. (2022). Friction Reduction of Oleamide Lubricants on Iron Surface. Drill. Fluid Complet. Fluid.

[25] Wang W. (2018). Design of Base Oil Molecular Structure for Diesel Engine and the Study of Lubricating Mechanism. Ph.D. Thesis.

[26] Zhang S., Yan Z., Liu Z., Jiang Y., Sun H., Wu S. (2023). Experimental and Numerical Study of the Mixed Lubrication Considering Boundary Film Strength. Materials.

[27] Khormali A., Ahmadi S. (2023). Prediction of Barium Sulfate Precipitation in Dynamic Tube Blocking Tests and Its Inhibition for Waterflooding Application Using Response Surface Methodology. J. Pet. Explor. Prod. Technol..

[28] Stephan S., Dyga M., Alhafez I.A., Lenhard J., Urbassek H.M., Hasse H. (2021). Reproducibility of Atomistic Friction Computer Experiments: A Molecular Dynamics Simulation Study. Mol. Simul..

[29] Liu D., Li H., Huo L., Wang K., Sun K., Wei J., Chen F. (2023). Molecular Dynamics Simulation of the Lubricant Conformation Changes and Energy Transfer of the Confined Thin Lubricant Film. Chem. Eng. Sci..

[30] Li T., Lei A., Ding F., Yang H., Hu G. (2017). Determination of Anti-wear Performance of Lubricant (four-ball machine method). https://www.plusstd.com/1551097592.html.

[31] Zhang X., Shan H., Li B., Zhang Y., Ji M., Cao Z., Liu J., Li Q. (2023). Technical requirements of non fluorescent biomass lubricant for drilling fluids. http://www.jxsyzj.com/product_view.asp?id=376.

